# An Ir(III) complex chemosensor for the detection of thiols

**DOI:** 10.1080/14686996.2016.1162081

**Published:** 2016-04-06

**Authors:** Zhifeng Mao, Jinbiao Liu, Tian-Shu Kang, Wanhe Wang, Quan-Bin Han, Chun-Ming Wang, Chung-Hang Leung, Dik-Lung Ma

**Affiliations:** ^a^ Department of Chemistry, Hong Kong Baptist University, Kowloon Tong, Hong Kong, P.R. China; ^b^ State Key Laboratory of Quality Research in Chinese Medicine, Institute of Chinese Medical Sciences, University of Macau, Macao, P.R. China; ^c^ School of Chinese Medicine, Hong Kong Baptist University, Kowloon Tong, Hong Kong, P.R. China

**Keywords:** Iridium(III) complex, chemosensor, thiols, 30 Bio-inspired and biomedical materials, 208 sensors and actuators

## Abstract

In this study, we report the use of a cyclometalated luminescent iridium(III) complex for the visualization of thiols. The detection of glutathione (GSH) by complex **1** is achieved through the reduction of its phendione N^N donor, which influences the metal-to-ligand charge-transfer (MLCT) of the complex. Complex **1** produced a maximum threefold luminescence enhancement at 587 nm in response to GSH. The linear detection range of **1** for GSH is between 0.2 and 2 M equivalents of GSH, with a detection limit of 1.67 μM. Complex **1** also displays good selectivity for thiols over other amino acids.

## Graphical abstract

**Figure:**
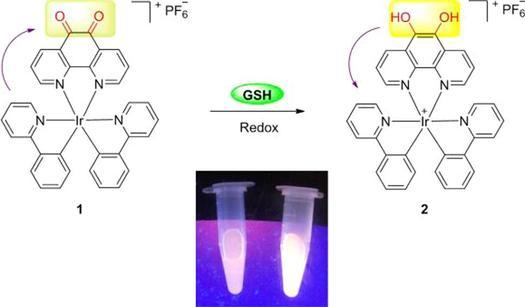

## 1. Introduction

Biological thiols play essential roles in cell function and maintenance. In particular, glutathione (GSH) is critically involved in redox homeostasis *in cellulo*. The dysregulation of GSH activity has been linked to diseases such as cancer, cystic fibrosis and neurodegenerative diseases.[1] Therefore, the development of sensitive detection methods for biothiols has recently been an active area of research.

Typical instrumental detection methods for biothiols include liquid chromatography,[2,3] capillary electrophoresis,[4] voltammetry [5] and flow injection.[6] However, those techniques require relatively complex sample preparation protocols and sophisticated instrumentation. Meanwhile, a number of chemosensors have been employed for the detection of thiols based on the thiol addition reaction, reviewed in [7,8]. However, thiol chemosensors are based on organic molecules,[9–17] and only a few examples of transition metal complexes as thiol chemosensors have been reported.

Compared with organic molecules, transition metal complexes are generally relatively easy to synthesize and modify, exhibit large Stokes shifts, and offer long-lived luminescence that could allow them to be potentially used in autofluorescent biological matrices.[18–24] Several iridium(III) complex chemosensors have been reported for thiol detection. Che and co-workers reported a FRET-based luminescent iridium(III) probe for the detection of cysteine (Cys) and homocysteine.[31] Later on some iridium(III) complexes were reported for thiol detection *in cellulo*.[32, 33] Li, Huang, Yi and co-workers have demonstrated iridium(III) complex chemosensors for selectively detecting homocysteine [34] or both homocysteine and cysteine,[35–38] and have employed these for the visualization of thiol in the cell. While Chao’s group reported an azobis(2,2′-bipyridine)-bridged dinuclear iridium(III) complex for the thiol imaging.[39] Additionally, Chen’s group reported an iridium(III) complex thiol chemosensor based on an α,β-unsaturated ketone motif.[40] In this project, we sought to employ an iridium(III) complex for the detection of thiols based on the redox reaction between GSH and the N^N donor (phendione) of a luminescent iridium(III) complex. We anticipate that the reduction of phendione by thiols would influence the metal-to-ligand charge-transfer (MLCT) state of the iridium(III) complex, thereby allowing the complex to function as a luminescent chemosensor for thiols detection.

## 2. Experimental details

### 2.1. Synthesis of [Ir(ppy)2(phendione)](PF6) 1

Complex **1** was reported in previous literature.[41] A suspension of [Ir(ppy)_2_]_2_Cl_2_ (ppy = 2-phenylpyridine) (0.2 mmol) and 1,10-phenanthroline-5,6-dione (phendione) (0.42 mmol) in a mixture of CH_2_Cl_2_:MeOH (1:1, 20 ml) was refluxed overnight under N_2_. The product mixture was then allowed to cool down to 25°C, and was filtered to remove unreacted dimer. To the filtrate, excess amount of NH_4_PF_6_ was added and the filtrate was reduced in volume by evaporation until precipitation of the crude product was observed. The precipitate was then filtered and washed by 40 ml water twice followed by 40 ml diethyl ether twice. The product was recrystallized by acetone: diethyl ether vapor diffusion to yield the titled compound as a brown solid.

## 3. Results and discussion

### 3.1. Design and synthesis of a thiols chemosensor

**Figure 1.**
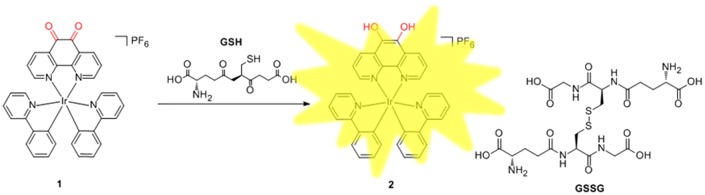
Mechanism of GSH detection by iridium(III) complex 1 while GSSG is oxidized by glutathione.

The photophysical properties of iridium(III) complex are sensitive to both the solvent environment and the nature of their C^N or N^N donor ligands. Phenanthrene-9,10-quinone, which has been associated with the production of reactive oxygen species (ROS), can be reduced back to catechol in futile redox cycles both enzymatically and nonenzymatically.[42] On the other hand, GSH, as the most abundant non-protein thiol, is a major reductant in internal cellular compartments.[15,43] As a consequence, we chose the structurally related 1,10-phenanthroline-5,6-dione (phendione) moiety as the N^N donor for the iridium(III) complex **1**,[41] which also coordinates two C^N ligands (ppy). We anticipated that the reduction of the phendione N^N donor by GSH, generating complex **2**, may influence the MLCT state of the iridium(III) complex, thereby allowing **1** to act as a luminescent chemosensor for thiols (Figure 1).

### 3.2. Photophysical properties of 1

We then examined the photophysical properties of complex **1**. Complex **1** displayed a 4.26 μs lifetime (Table S1), which is in the same order as those exhibited by phosphorescent transition metal complexes, while organic chemosensors generally exhibit lifetimes in the nanosecond range. This demonstrates the benefit of using transition metal complexes as chemosensors, in which their long-lived luminescence could potentially allow their emission to be identified from a strongly autofluorescent background signal by utilizing time-resolved luminescence spectroscopy. Moreover, **1** exhibited a maximum emission wavelength at 587 nm upon excitation at 350 nm. The Stokes shift is approximately 237 nm, which is much higher than those typically exhibited by organic chemosensors.

### 3.3. Signal response of 1 to GSH

**Figure 2.**
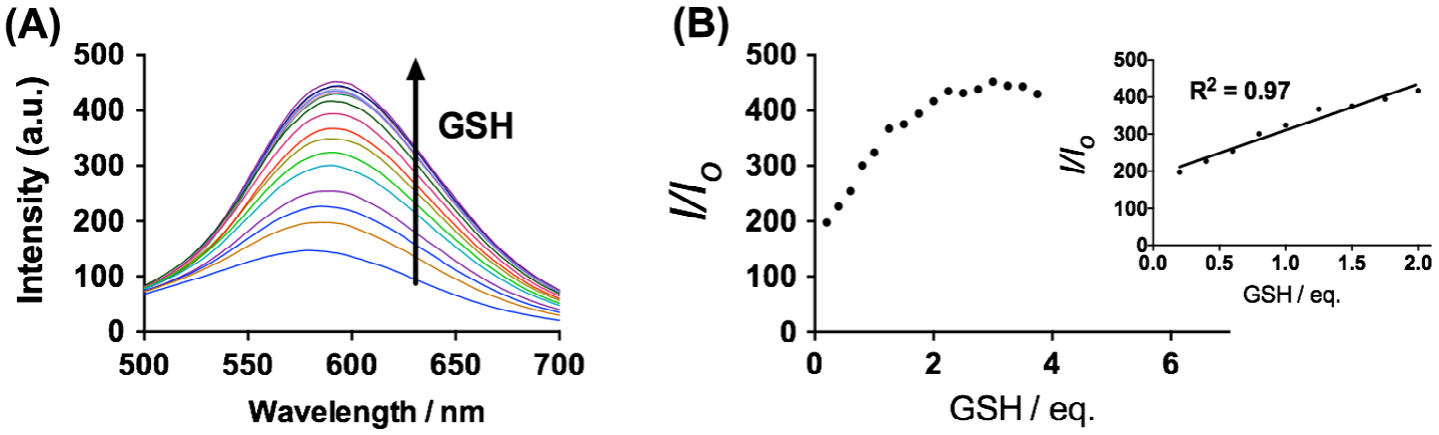
(A) Luminescence spectra of **1** (20 μM) with increasing concentration of GSH (0–3.75 eq.) in DMSO:HEPES 9:1 (10 mM, pH 7.0). (B) The relationship between luminescence intensity and GSH concentration.

**Figure 3.**
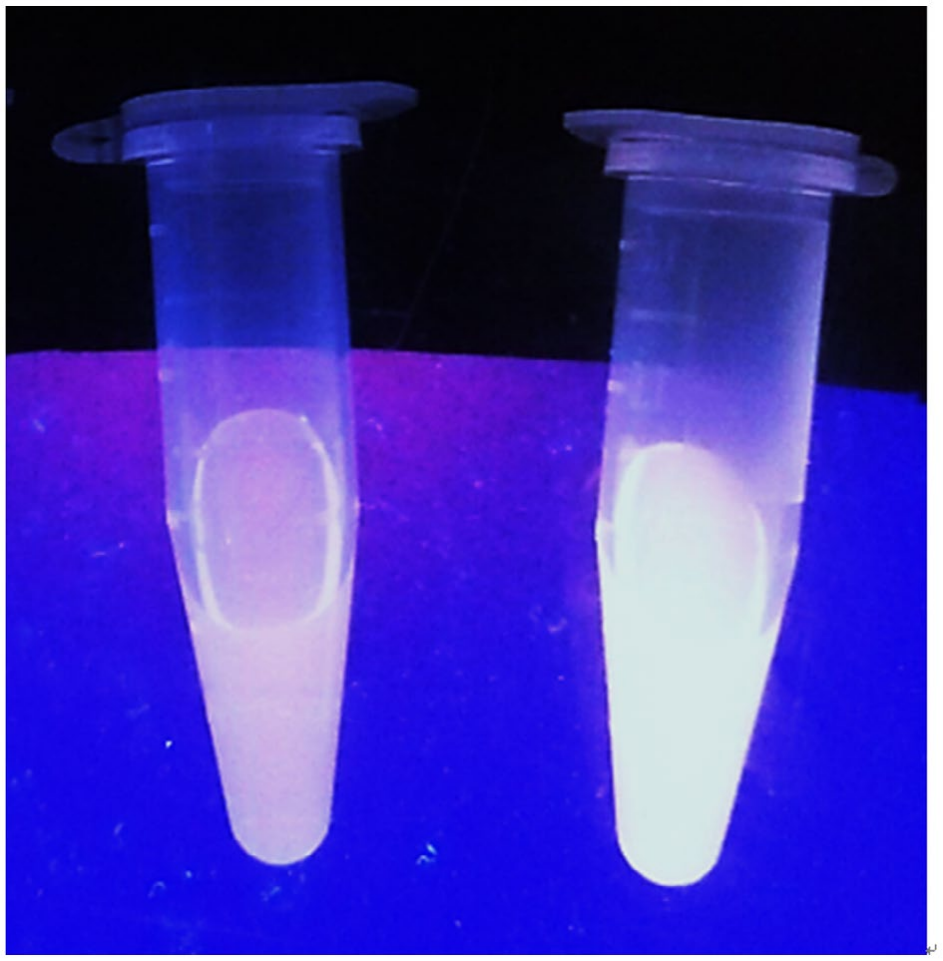
Photographs of **1** (20 μM) with (left) and without (right) 20 μM GSH under UV illumination.

We next examined the emission response of **1** towards GSH. In the absence of GSH, the luminescence intensity of **1** is weak in a 9:1 mixture of dimethyl sulfoxide and 4-(2-hydroxyethyl)-1-piperazineethanesulfonic acid (DMSO:HEPES, 10 mM, pH 7.0). However, upon addition of GSH, a significant enhancement in the luminescence intensity of **1** was recorded. The luminescence of **1** (20 μM) increased with increasing concentration of GSH and was saturated at two molar equivalents of GSH, with a *ca.* threefold enhancement (Figure 2). A linear relationship (*R* ^2^ = 0.99) was measured in the range of 0.2‒2 M equivalents of GSH, while the limit of detection at a signal-to-noise ratio of 3 was calculated to be 1.67 μM, which is sufficient for detecting GSH in blood (in micro molar levels).[44] Moreover, the presence of GSH is transduced by **1** into an observable signal that could be observed by the naked eye upon UV illumination (Figure 3).

### 3.4. Mechanism validation

**Figure 4.**
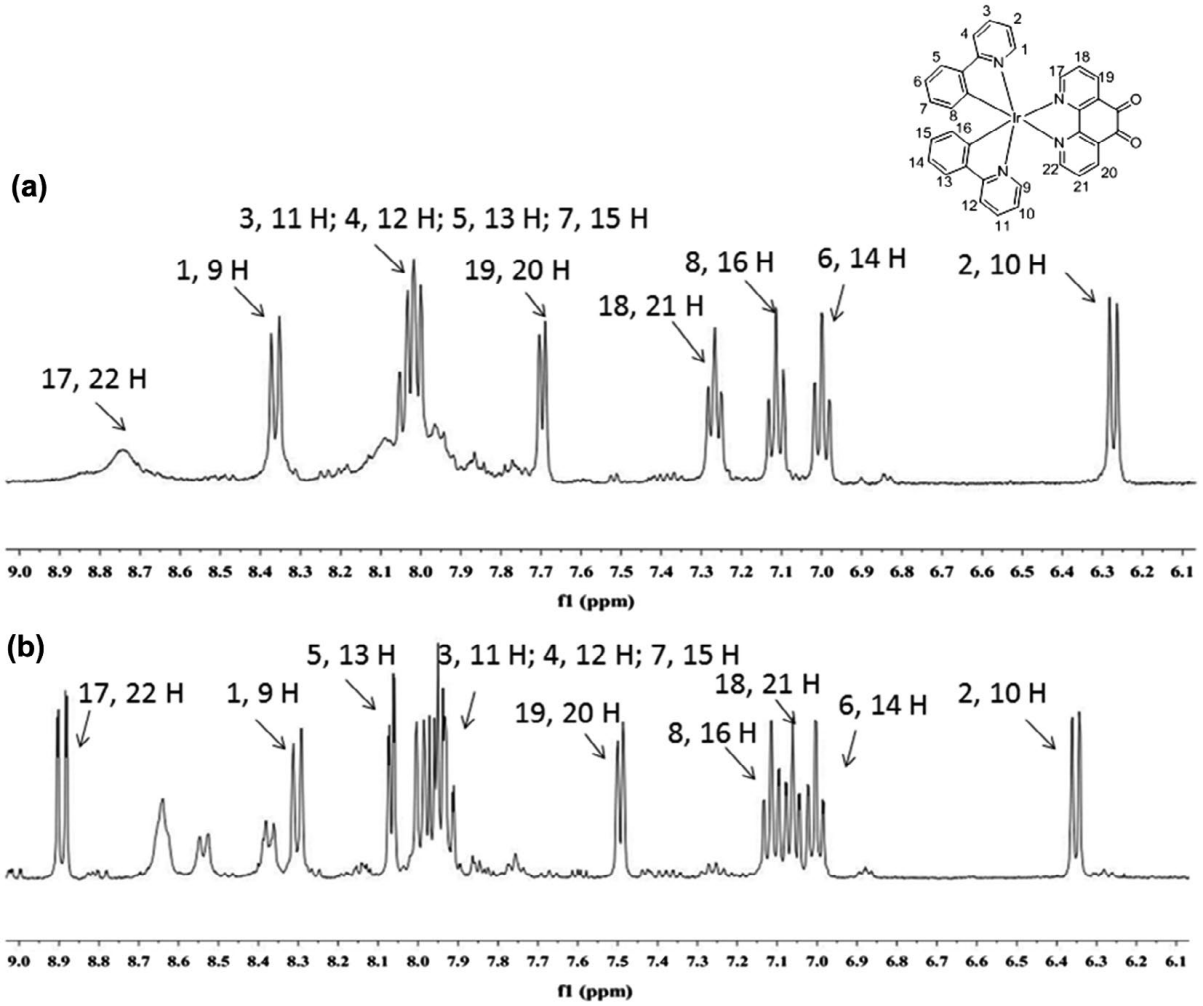
Partial ^1^H NMR spectra of **1** (20 μM) upon the addition of GSH (2 M equivalents) in DMSO-*d*_6_. (a) **1**; (b) **1**+GSH.

**Figure 5.**
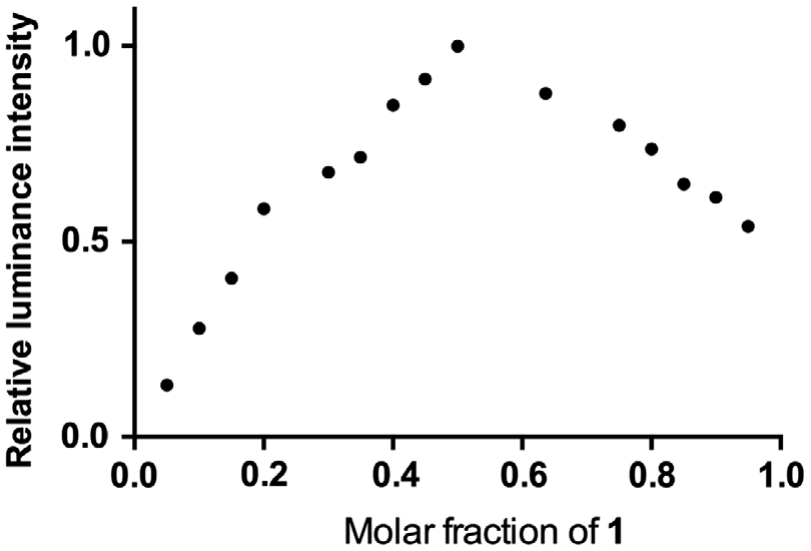
Job’s plot of both **1** and GSH in DMSO:HEPES 9:1 (10 mM, pH = 7.0). Total concentration 5 μM, emission measured at 587 nm.

To verify our hypothesis that GSH will reduce the non-emissive complex **1** to form the emissive complex **2**, the reaction of **1** with GSH was measured by ^1^H nuclear magnetic resonance spectroscopy (NMR, Figure 4). Upon the addition of GSH, the signals of several protons of the phenanthroline ring were signiﬁcantly shifted. Moreover, high-resolution mass spectrometry analysis of the product mixture revealed the formation of **2** at m/z = 713.1512 (Figure S2), while the expected signal for complex **1** at m/z = 711.1399 was diminished (Figure S3). Furthermore, Job’s plot analysis was employed to study the binding stoichiometry of the iridium(III) complex **1** with GSH. The highest luminescence intensity of complex **1** was achieved at a mole fraction of approximately 50% GSH (Figure 5), suggesting that a 1:1 ratio is the most possible binding stoichiometry of complex **1** with GSH. While 1:1 binding in Job’s plot may suggest that radical anion of **1** which prepared by one electron reduction is as emissive as **2** that presumably useful for the extension to radical/H_2_O_2_ sensors.

### 3.5. Selectivity of iridium(III) complex 1 for GSH

**Figure 6.**
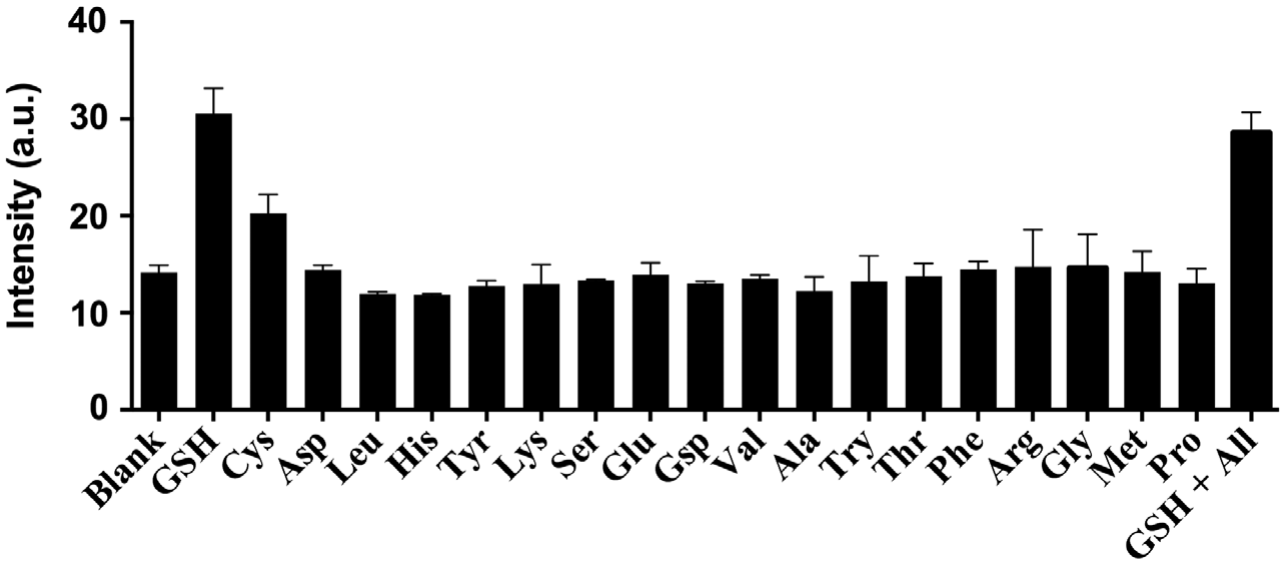
Luminescence response of 20 μM **1** with 0.8 M equivalents of GSH and Cys or 1.0 M equivalent of other amino acids in DMSO:HEPES 9:1 (10 mM, pH = 7.0).

As selectivity is an important parameter for a chemosensor, we evaluated the selectivity of **1** by introducing 0.8 M equivalents of GSH and Cys or 1 M equivalent of common amino acids into a solution of **1** (20 μM) (Figure 6). Encouragingly, the luminescence response of **1** towards GSH and Cys was significantly stronger than that of 1 M equivalent of other amino acids while the luminescence enhancement of GSH is twofold higher than that of Cys. This is presumably due to the different oxidizing ability. These results demonstrate the selectivity of the chemosensor **1** for thiols over amino acids. We also performed a competition experiment to investigate the response of **1** towards GSH and (1 M equivalent) in the presence of a mixture of all interfering amino acids (1 M equivalent each). Encouragingly, the luminescence intensity of **1** was not significantly affected upon the addition of different interfering amino acids, indicating that **1** could possibly be utilized to detect thiols in a real sample matrix in the presence of other amino acids.

## 4. Conclusions

In conclusion, we have employed the iridium(III) complex **1** for the detection of thiols. We postulate that the reduction of the phendione N^N donor by thiols may influence the MLCT state of complex **1**, thereby enabling **1** to function as a luminescent chemosensor for thiols. The proposed mechanism of the chemosensor was supported by NMR and high-resolution mass spectrometry analysis. Complex **1** produced a maximum threefold luminescence enhancement at 583 nm in response to GSH. The linear detection range of **1** for GSH is 0.2‒2 M equivalents of GSH, with a detection limit of 1.67 μM. Complex **1** also displays good selectivity for GSH and Cys over common amino acids. Compared with common organic chemosensors, **1** displays a large Stokes shift and a long-lived luminescence that may favor its use in strongly autofluorescent biological samples.

## Disclosure statement

No potential conflict of interest was reported by the authors.

## Funding

This work is supported by Hong Kong Baptist University FRG2/14-15/004 and FRG2/15-16/002), the Health and Medical Research Fund [HMRF/14130522], the Research Grants Council [HKBU/201811, HKBU/204612 and HKBU/201913], the French Agence Nationale de la Recherche/Research Grants Council Joint Research Scheme (A-HKBU201/12; Oligoswitch ANR-12-IS07-0001), National Natural Science Foundation of China [21575121], Guangdong Province Natural Science Foundation [2015A030313816], Hong Kong Baptist University Century Club Sponsorship Scheme 2015, Interdisciplinary Research Matching Scheme [RC-IRMS/14-15/06], the Science and Technology Development Fund, Macao SAR [098/2014/A2], the University of Macau [MYRG091(Y3-L2)-ICMS12-LCH, MYRG2015-00137-ICMSQRCM, MRG023/LCH/2013/ICMS and MRG044/LCH/2015/ICMS)].
